# High Current Anxiety Symptoms, But Not a Past Anxiety Disorder Diagnosis, are Associated with Impaired Fear Extinction

**DOI:** 10.3389/fpsyg.2016.00252

**Published:** 2016-02-26

**Authors:** Puck Duits, Danielle C. Cath, Ivo Heitland, Johanna M. P. Baas

**Affiliations:** ^1^Department of Clinical and Health Psychology, Utrecht UniversityUtrecht, Netherlands; ^2^Altrecht Academic Anxiety CentreUtrecht, Netherlands; ^3^Department of Experimental Psychology, Utrecht UniversityUtrecht, Netherlands; ^4^Helmholtz Research InstituteUtrecht, Netherlands

**Keywords:** anxiety, extinction, treated patients, cue, context, startle, fear conditioning

## Abstract

Although impaired fear extinction has repeatedly been demonstrated in patients with anxiety disorders, little is known about whether these impairments persist after treatment. The current comparative exploratory study investigated fear extinction in 26 patients treated for their anxiety disorder in the years preceding the study as compared to 17 healthy control subjects. Fear-potentiated startle and subjective fear were measured in a cue and context fear conditioning paradigm within a virtual reality environment. Results indicated no differences in fear extinction between treated anxiety patients and control subjects. However, scores on the Beck Anxiety Inventory across all participants revealed impaired extinction of fear potentiated startle in subjects with high compared to low anxiety symptoms over the past week. Taken together, this exploratory study found no support for impaired fear extinction in treated anxiety patients, and implies that current anxiety symptoms rather than previous patient status determine the success of extinction.

## Introduction

Fear extinction serves as a potential model to explain the development, maintenance and treatment of anxiety disorders, and extinction of fear has been studied extensively in patients with anxiety disorders over the past decades. Support for the role of impaired fear extinction in the development of post-traumatic stress disorder was found in prospective studies, in which stronger fear responses during extinction predicted the onset of posttraumatic stress symptoms at a later stage (Guthrie and Bryant, 2006; Orr et al., 2012; Lommen et al., 2013). We recently published a meta-analysis that provided evidence for the role of impaired fear extinction in patients with a current anxiety disorder (Duits et al., 2015). Robustly higher fear responses to danger cues (CS+’s) and stronger differentiation between danger and safety cues (CS+ and CS–) were demonstrated in patients with anxiety disorders compared to healthy subjects during extinction (Duits et al., 2015). Support for the predictive value of fear extinction on treatment outcome was found in recent studies conducted in patients with panic disorder and agoraphobia (Kircher et al., 2012; Lueken et al., 2013; Hahn et al., 2014). Treatment non-responders displayed enhanced activation of threat-related brain systems in response to safety cues during extinction when compared to responders (Lueken et al., 2013).

Although many studies have investigated differences in fear extinction between patients with anxiety disorders and healthy control subjects, little is known about whether impaired extinction of fear persists in patients who have been treated for an anxiety disorder earlier in their life. Persistent reduced extinction of fear would suggest that performance on extinction tasks represents an endophenotypic trait rather than a temporary condition. Indeed, individual differences in fear extinction seem to be moderately heritable (Hettema et al., 2003) and fear acquisition (Zeidan et al., 2012; Torrents-Rodas et al., 2014) and extinction (Fredrikson et al., 1993) parameters seem to be relatively stable over time. The relative stability of conditioning parameters supports the idea that patients with an anxiety disorder will not only demonstrate impaired extinction of fear at the time of their illness, but also after treatment and later in life. However, on the other hand, some studies suggest that brain mechanisms underlying fear extinction might strengthen over time as a consequence of exposure-based cognitive behavioral treatment in patients with anxiety disorders, suggesting changeability of these mechanisms related to fear extinction mechanisms (Kircher et al., 2012; Galvao-de et al., 2013).

The current study aimed to explore differences in the extinction of fear responses between treated anxiety patients and healthy control subjects. Startle responses as well as subjective fearfulness were measured to assess whether any group differences exist in cue-specific fear responses (as previously demonstrated by Lissek et al., 2005 and Duits et al., 2015), contextual-specific fear responses (Grillon et al., 2008, 2009) and generalization of fear during extinction. In accordance with a dimensional transdiagnostic approach (as initiated by the NIMH in their Research Domain Criteria; RDoC; Insel et al., 2010), we assessed fear responding as well as current anxiety symptoms as continuous variables across two closely related diagnostic groups (see Materials and Methods) and a control group. Considering that fear extinction has been shown to be partly genetically determined and relatively stable over time, we hypothesized that patients who have been treated for an anxiety disorder in the past would still show stronger fear responses during extinction learning to both the threat cue and threat context, and show stronger generalization of fear responses, as compared to healthy control subjects. This paper focusses on the extinction of fear, but fear acquisition data are also reported as potential differences in fear acquisition may confound subsequent analyses on the extinction of fear.

## Materials and Methods

### Participants

Thirty anxiety patients were recruited via Altrecht Academic Anxiety Centre in Utrecht, a specialized outpatient clinic for anxiety disorders. Four patients were excluded from further analyses, as these patients used benzodiazepines and/or antipsychotics at time of participation, which can substantially influence fear responses during the conditioning procedure (Baas et al., 2002; Brignell and Curran, 2006). Patients had been primarily diagnosed with either a panic disorder with agoraphobia (*N* = 13) or a generalized social anxiety disorder (*N* = 13). Both panic disorder with agoraphobia and social anxiety disorder belong to the main types of phobic disorders (Antony and Swinson, 2000). We investigated these two types of phobic disorders concomitantly (Liberman et al., 2006; Lau et al., 2008; Reeb-Sutherland et al., 2009; Waters et al., 2009), as the underlying theory assumes that delayed and/or reduced extinction of fear cuts across the diagnostic categories of phobic disorders. All diagnoses had been established prior to treatment according to DSM-IV classification criteria (American Psychiatric Association, 2000), with the aid of the Structured Clinical Interview for DSM-IV-TR Axis I Disorders (First et al., 2002). Ninety-two percent (*n* = 24) of the patients had a comorbid diagnosis, see Supplementary Table S1 for a list of comorbid diagnoses in the patient group before therapy.

All included patients had received exposure therapy with response prevention (ERP) at the outpatient clinic between 2008 and 2012. In 12 patients ERP was combined with serotonin reuptake inhibitors. Fourteen of the included patients were medication-free, one patient used methylphenidate combined with a serotonin-reuptake inhibitor.

Time period between treatment and participation in the fear conditioning experiment of this study ranged between 2 months and 4 years (*M* = 1.4 years, *SD* = 1.1). ERP (with or without pharmacotherapy) encompassed an average of 18 (*SD* = 8) 45-min sessions. Treatment focused on reducing panic or social anxiety symptoms and avoidance behavior by cognitive therapeutic techniques, interoceptive exposure, and exposure to the feared situations.

Treatment outcome in patients with panic disorder and agoraphobia was measured using the Body Sensations Questionnaire (BSQ; Chambless et al., 1984). On average, patients with panic disorder and agoraphobia demonstrated 21% improvement of their anxiety symptoms (measured using BSQ mean scores) between pre- and post-treatment, and 11% improvement when comparing pre-treatment symptoms with symptoms at the time of participation in the current study. Six patients were in full remission, with scores below the cutoff criterion of 1.93 (using the BSQ), at time of participation and at time of post-treatment measurement (de Beurs, 1993). The Social Phobia and Anxiety Inventory 18-item version (SPAI-18; Beidel et al., 1989) was used to assess treatment outcome in patients with social anxiety disorder. Patients with social phobia showed on average 14% improvement between pre-treatment and post-treatment, and 26% improvement when comparing pre-treatment symptoms with symptoms at time of participation in the current study. Based on a cutoff criterion of 48 (de Vente et al., 2014), treatment effects were clinically significant in four treated patients with social anxiety disorder (31%) at time of participation in the current study and in three patients (23%) at time of post-treatment.

Twenty age-, sex-, and education-matched healthy control subjects (see **Table 1**) were recruited through advertisements in supermarkets and community centers. Healthy controls were free of psychotropic medication, and were screened on the absence of lifetime axis I diagnoses using the Mini International Neuropsychiatric Interview (Van Vliet and De Beurs, 2007). Three control subjects (and none of the patients) were excluded as they failed to indicate in which context the shocks were administered during uninstructed acquisition (see also Supplementary Material). The final sample consisted of 17 healthy control subjects. Exclusion of the three context-unaware control subjects did not significantly alter the results of the current study.

**Table 1:** Demographic characteristics of the patient and control group.

	Patient group (*N* = 26)		Control group (*N* = 17)		Significance of group differences^a^
	*N*	%	*N*	%	
Male	19	73.1	13	76.5	NS
Female	7	26.9	4	23.5	NS
Medication	12	46.2	0	0	*p* = 0.001
	**Mean**	***SD***	**Mean**	***SD***	
Age	34.7	9.5	35.2	10.2	NS

^a^*Chi-square tests (except for age, which was assessed using a two-tailed independent samples t-tests).*

Experimental procedures were approved by the Medical Research Ethics Committee of the University Medical Centre Utrecht. Participants were informed about the current study by telephone and by means of an information letter. Subsequently, all participants were asked whether they had hearing problems, to ensure that participants would respond to the startle probes in the experiment. Written informed consent was given by all participants, and all subjects received a financial compensation for their time while participating in the experiment (between aaa 25,– and aaa 35,–).

### Symptom Ratings at Time of Testing

The following questionnaires were completed by all subjects to measure presence and severity of symptoms of anxiety and depression at testing: the Beck Anxiety Inventory (BAI; Beck et al., 1988), the Anxiety Sensitivity Index (ASI; Peterson and Heilbronner, 1987; Rodriguez et al., 2004), and the Beck Depression Inventory – II (BDI; Beck et al., 1961). The BAI questionnaire (containing 21 items) focusses mainly on physiological symptoms of anxiety that have been present over the past week including the day of participating in the study (Beck et al., 1988), while the 16-item ASI assesses a subjects’ belief about the consequences of their anxiety symptoms. The trait subscale of the State-Trait Anxiety Inventory (STAI; Spielberger et al., 1983) was included to assess trait anxiety. Patients formerly diagnosed with panic disorder and agoraphobia completed the BSQ (Chambless et al., 1984), while patients with social anxiety disorder filled out the SPAI-18 (Beidel et al., 1989) to follow them on specific symptom change. Control subjects completed both the BSQ and SPAI-18 to optimize comparability with the patient group at testing (**Table 2**).

**Table 2:** Symptom ratings in the patient and control group.

	Patient group (*N* = 26)		Control group (*N* = 17)		Significance of group differences^a^
	Mean	*SD*	Mean	*SD*	
Anxiety Sensitivity Index (ASI)	17.4	10.4	5.8	4.5	*p* < 0.001
Beck Anxiety Inventory (BAI)	12.2	8.8	4.9	4.7	*p* = 0.008
Beck Depression Inventory (BDI)	12.6	10.4	6.2	7.4	*p* = 0.036
STAI – trait	48.4	7.4	41.2	5.6	*p* = 0.004
BSQ at testing	2.2	0.9	1.4	0.4	*p* = 0.010
BSQ *before treatment*	2.7	0.7	–	–	NA
SPAI-18 at testing	54.8	13.9	34.6	15.2	*p* = 0.001
SPAI-18 *before treatment*	73.8	10.0	–	–	NA

^a^*Two-tailed independent samples t-tests.*

### The Conditioning Procedure

**FIGURE 1:**
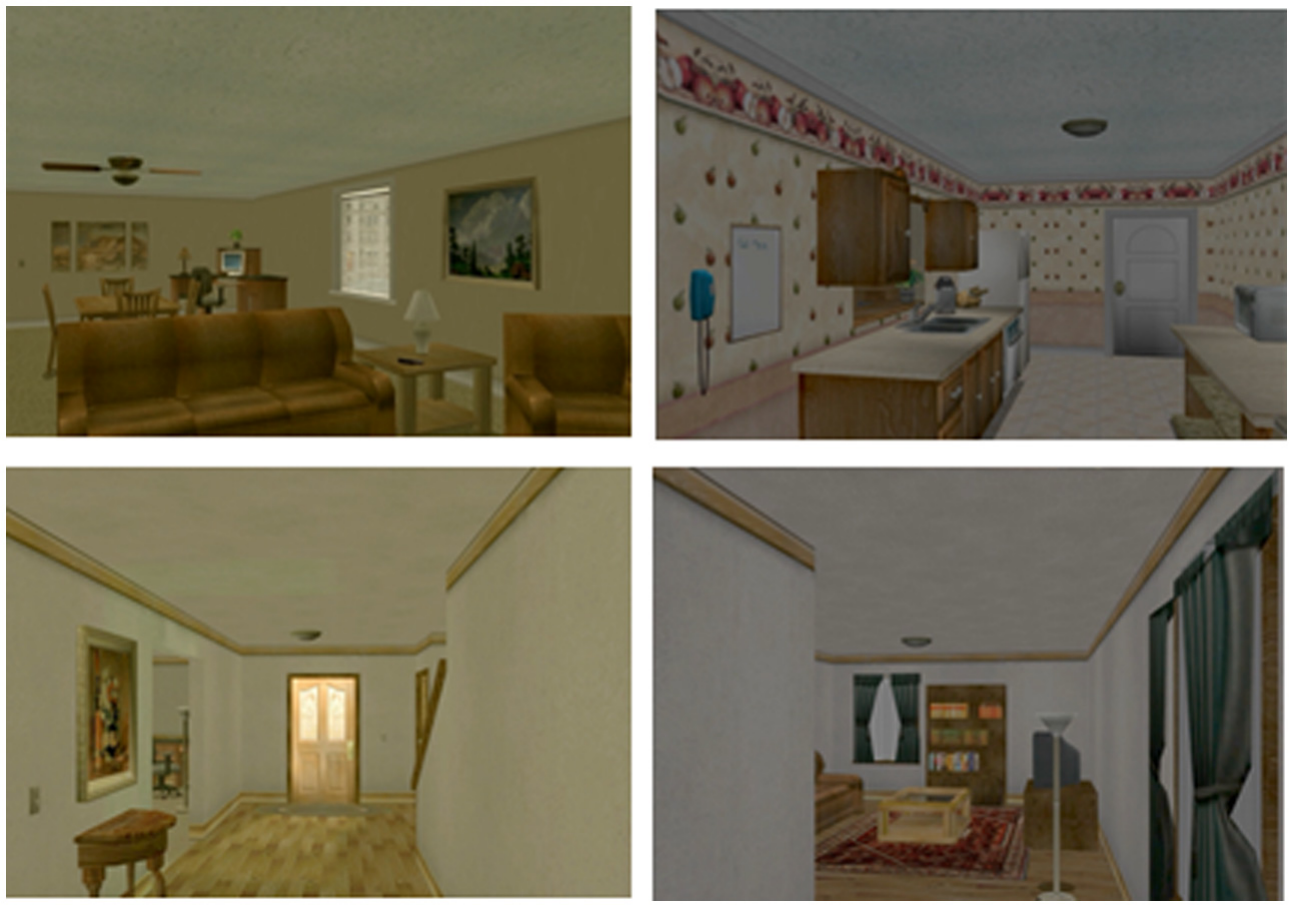
**Examples of screenshots from the fear conditioning task**. The upper two screenshots show the apartment with the light on and off. The lowest screen shots present the suburban house with the light on and off.

The experimental procedure was conducted in a sound-insulated laboratory with dimmed light. Participants were seated in front of a computer screen and surface electrodes were attached to measure the eyeblink startle reflex. We used the well-established virtual reality fear conditioning task developed by Baas et al. (2004, 2008), Heitland et al. (2012), and Baas (2013), with the same design as in Heitland et al. (2012). Participants viewed prerecorded movies in a virtual environment consisting of scenes from two different contexts: an apartment and a suburban house. The conditioned stimulus (CS) consisted of an 8-s duration subtle increase in background illumination. The CS was presented four times in each context per block. In one context (threat; counterbalanced across subjects), presentation of the CS could be paired with the unconditioned stimulus (US), which was an electrical shock. The US was presented co-terminating with the CS offset and never at other times. In the other context (safe), presentation of the CS was never paired with the US. This created four different conditions: shock context light on, shock context light off, safe context light on and safe context light off (see **Figure 1**). The two contexts were connected to each other by short scenes in the metro and the street. A shock work up procedure was completed prior to the conditioning task to establish an individual shock level that was perceived as ‘quite annoying but not painful’ by each participant. Subjective ratings of shock aversiveness were obtained after each block.

**FIGURE 2:**
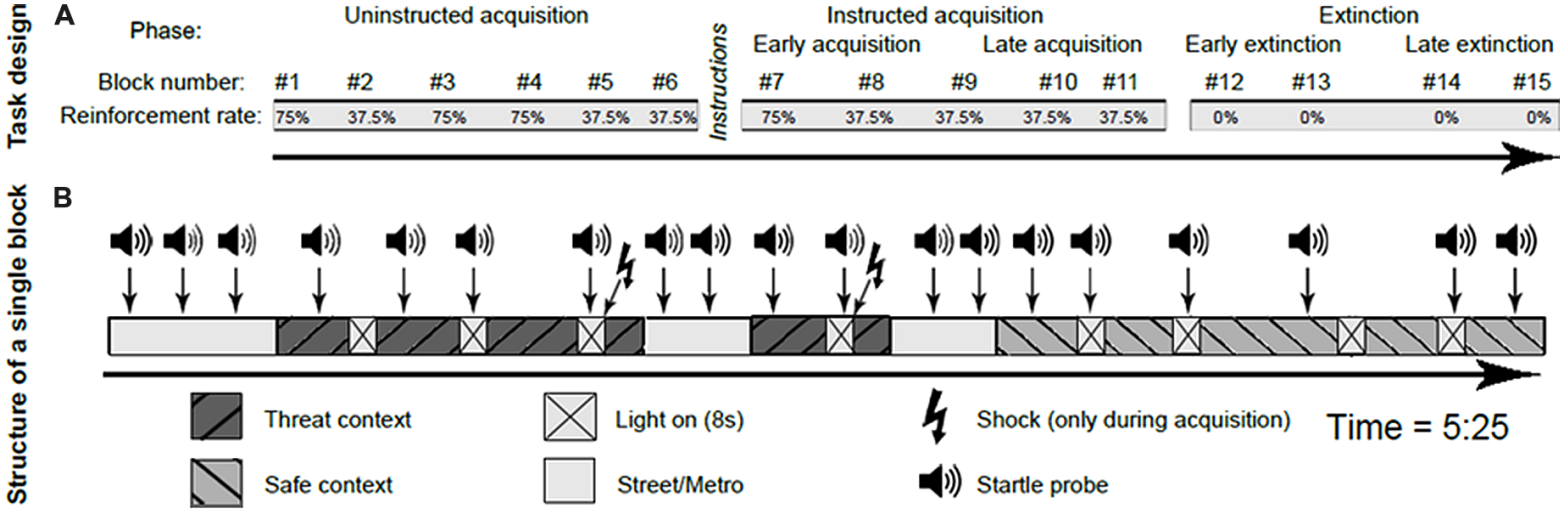
**Schematic representation of the conditioning paradigm, including the task design (A) and the structure of a single block (B)**. This figure is adapted from Heitland et al. (2013).

The fear conditioning task consisted of 16 blocks. Each block lasted 5.25 min and started with a short scene in the metro and street in which three startle probes were presented for habituation of the startle reflex. All blocks included one long visit to the shock or safe context and two short visits to the shock or safe context (Baas et al., 2008). There were four CS presentations in each context, regardless of whether the context was visited once (duration was 90 s, including four CS presentations) or twice (visit durations were 70 s and 30 s, including three and one CS presentation, respectively). In each block, during three out of four CS presentations per context a startle probe was presented, as well as three probes in absence of the CS in each context (see **Figure 2**). Scenes of the metro and the street were displayed for 40 s during the transition between visits. Each metro/street scene contained two startle probes, to prevent dishabituation of the startle.

Similar to Heitland et al. (2012), the experiment consisted of four distinct phases: habituation (one block), uninstructed acquisition (six blocks), instructed acquisition (five blocks), and extinction (four blocks). The habituation phase consisted of 12 startle probe presentations, followed by a video to get acquainted to the virtual environment. Prior to the uninstructed acquisition phase, subjects were told that from now on, they could receive shocks and that they might be able to predict the onset of these shocks by watching the movie scenes carefully. This uninstructed acquisition phase was designed to measure the spontaneous learning of cue and context associations with the shock occurrence. At the end of the uninstructed acquisition phase, all subjects were asked to report on what they considered to predict the onset of the shock. Groups did not differ in contingency awareness (additional data in Supplementary Table S2). At the start of the instructed acquisition phase, subjects received an explicit verbal and written on-screen instruction in which the specific contingency between danger context, CS, and US was described. The instructed phase was included in the paradigm to assess responses once contingencies were acquired, and to ensure contingency learning before extinction started. During both acquisition phases, some blocks (1, 3, 4, and 7) contained relatively high (75%) reinforcement rates to ensure acquisition, while other blocks (2, 5, 6, 8, 9, 10, and 11) contained low (37,5%) rates of reinforcement. During the low reinforcement blocks, the shock was given during the last CS of that visit (i.e., the first, third or fourth CS, depending on whether it relates to a long or two short visits of the shock context within that block). Blocks with low reinforcement rates were introduced to minimize the effects of shock sensitization on physiological responding, which is especially relevant for the unbiased assessment of context responses (Baas et al., 2008; Baas, 2013). Psychophysiological measures are only reported from the blocks with the low reinforcement rates, as in these blocks startle probes do not follow directly upon a shock presentation in the shock context, and the safe context is visited equally often as the shock context after shock reinforcement. The extinction phase, in which the CS was no longer paired with the US in the shock context, followed immediately after the instructed acquisition and started without explicit instructions.

### Stimuli and Physiological Apparatus

Electrical shocks were administered by a Digitimer DS7A generator. The shock electrodes were applied over the medial nerve on the inner left wrist. A train of 125 pulses of 2-ms length served as US, lasting for 625 ms.

The startle probes were 50-ms, 105 dB (A) white-noise bursts delivered through headphones (Sennheiser Electronic HD 202). The electromygraphic (EMG) activity associated with each startle trial was recorded with two Ag/AgCl electrodes on the orbicularis oculi under the right eye, using a Biosemi ActiveTwo system (BioSemi Instrumentation, Amsterdam, The Netherlands).

### Subjective Fear Ratings

Subjective fearfulness ratings were obtained after each block during uninstructed acquisition, instructed acquisition, and extinction. The measurement was conducted using visual analog scales (VAS) that were presented on the computer. Screenshots from movie clips (randomly drawn from a set of 10 per condition, see **Figure 1** for examples) were presented simultaneously with the VAS scale, with the instruction to rate the level of fear associated with the given situation [anchors were ‘not at all fearful of shock’ (0) and ‘very fearful of shock’ (100)]. Each condition was presented twice (using different screenshots) after every block to allow a reliable assessment of subjects’ fearfulness ratings per condition.

### Data Reduction

Brain Vision Analyzer software was used to process the startle data according to published guidelines and previous studies (Blumenthal et al., 2005; Baas et al., 2008; Baas, 2013). For detailed information regarding startle processing and identification of artifacts and null responses, see Böcker et al. (2004). Potential differences in baseline startle amplitudes and shock intensity between treated anxiety patients and control subjects were investigated first, as these were regarded as potential confounds during extinction. For all subsequent analyses, startle amplitudes were transformed into *z*-scores = (raw score – mean of all startle trials within each subject)/standard deviation to control for individual pre-existing differences in baseline startle amplitudes. Thereafter, mean startle amplitudes were computed per condition per block (average of three raw startle amplitudes). Only startle data from the acquisition blocks with low (37.5%) reinforcement shock rates (blocks 2, 5, 6, 8, 9, 10, 11), and all extinction blocks (blocks 12, 13, 14, 15) were included in the analyses to prevent contamination of the data by shock sensitization. Participants’ physiological data were included in the final analysis if at least one artifact-free trial for each block was recorded per stimulus type. Startle data from one control subject was missing in the final analyses.

### Statistical Analyses

Analyses were consistent with previous, similar fear conditioning studies (Heitland et al., 2012, 2013), and were conducted with IBM SPSS Statistics (version 20.0). Fear potentiation was studied using cue-specific, context-specific, and generalization contrasts. First, cue-specific fear responses were calculated by subtracting fear responses to the shock room light vs. shock room dark. Second, context-specific fear responses were computed by contrasting fear responses to the shock room dark vs. safe room dark. Third, generalization of fear was calculated by subtracting shock room light vs. safe room light. Startle and subjective outcome measurements were analyzed separately per phase (uninstructed acquisition, instructed acquisition, and extinction) using repeated measures General Linear Model (GLM) Analyses of Variance (ANOVAs). To assess the reduction of fear potentiation of startle and VAS responding during extinction, late acquisition (the last two blocks during instructed acquisition) was contrasted to late extinction (blocks 14 and 15; where the effect of extinction over time becomes apparent). Group, defined as treated anxiety patients vs. healthy controls, was included in the ANOVA’s as between-subjects factor. Within-subject factors were block and cue-specific fear-potentiated startle (FPS)/context-specific FPS/generalization FPS.

#### Follow-Up Analyses

Subsequently, in case of a significant interaction between cue-specific FPS/context-specific FPS/generalization FPS and group, follow up analyses were conducted per contrast to determine the nature of the effect. First, the change in fear-response over time was examined per group, to assess whether acquisition or extinction learning took place within each group. Second, differences in fear responding were tested between groups during the first and last two blocks of the phase, to investigate within-phase changes in fear responding.

#### Covariates

Medication use was added as between-subjects variable in all GLM analyses described above, to control for (1) the unequal distribution of medication use between groups and (2) potential effects of medication on fear conditioning. In addition, we controlled for individual differences in anxiety symptoms at time of participation, because higher rates of anxiety symptoms may lead to stronger fear responses during extinction (Grillon et al., 1993; Jackson et al., 2006; Vriends et al., 2010). Moreover, anxiety symptoms at the time of participating may differ substantially across patients treated for an anxiety disorder, because the time between treatment and participating in the current paradigm was not standardized. The BAI (Beck et al., 1988) was included as continuous covariate to control for differences in anxiety symptoms at time of participation. In case a significant interaction between BAI and cue-specific FPS/context-specific FPS/generalization FPS was found in the main analyses, follow up analyses were conducted to determine the nature of these interaction effects and to allow visualization of the effects. Therefore, participants were categorized as subjects with high (BAI > 9) vs. low (BAI 0–9) anxiety symptoms over the past week. The cut-off to index anxiety symptoms at testing (BAI = 9) was based on a median split, which corresponds to the cut score that has been recommended by other studies that investigated psychometric properties of the BAI and the utility of this questionnaire as a screener for anxiety disorders (Kabacoff et al., 1997; Leyfer et al., 2006). High (*N* = 15) as well as low (*N* = 10) BAI scores were observed in the treated patient group. Furthermore, 10 control subjects reported low anxiety symptoms over the past week, while three control subjects had high anxiety symptoms. BAI scores from one patient and four control subjects were missing. See Supplementary Table S3 for the distribution of clinically relevant anxiety symptoms (based on cut off scores from symptom specific questionnaires) across subjects with high vs. low anxiety symptoms.

#### Additional Analyses

Last, GLM ANOVA’s were also conducted to investigate differences in fear extinction between treatment responders vs. non-responders. Cutoff scores from the BSQ and SPAI-18 were used for the grouping of responders and non-responders. No significant group differences in fear conditioning were found between responders (*N* = 7) and non-responders (*N* = 18). Data pertaining to this line of analyses is available upon request.

## Results

### Shock Work Up

Group differences in shock intensity were tested to assess whether this basic parameter could account for group differences. Independent samples *t*-tests demonstrated no significant differences in the final shock intensity that was established after the shock work up between treated anxiety patients (*M* = 1.5 mA, *SD* = 0.6) and controls (*M* = 1.9 mA, *SD* = 1.1), *t*(41) = –1.09, *p* = 0.286, *d* = –0.45. Furthermore, a repeated measures ANOVA demonstrated no significant difference in self-reported VAS ratings of shock aversiveness (measured after each block) between treated anxiety patients and controls (main effect group: *F*(1,41) = 1.4, *p* = 0.244, $\eta_{p}^{2}=0.03$; group by stimulus interaction: *F*(4.6,190.3) = 0.8, *p* = 0.556, $\eta_{p}^{2}=0.02$).

### Baseline Startle Amplitude

Group differences in baseline startle amplitudes were investigated to control for pre-existing group differences prior to the conditioning procedure. Results demonstrated significantly higher baseline startle amplitudes in the treated patient group (*M* = 114.1 μV, *SD* = 50.1), as compared to controls during startle habituation series (*M* = 74.9 μV, *SD* = 44.), *F*(1,41) = 7.1, *p* = 0.011, $\eta_{p}^{2}=0.15$. Analyses of the FPS during conditioning were performed with *z*-transformed values to correct for individual baseline differences (see Data Reduction).

### Startle

#### Startle – Task Effects

**FIGURE 3:**
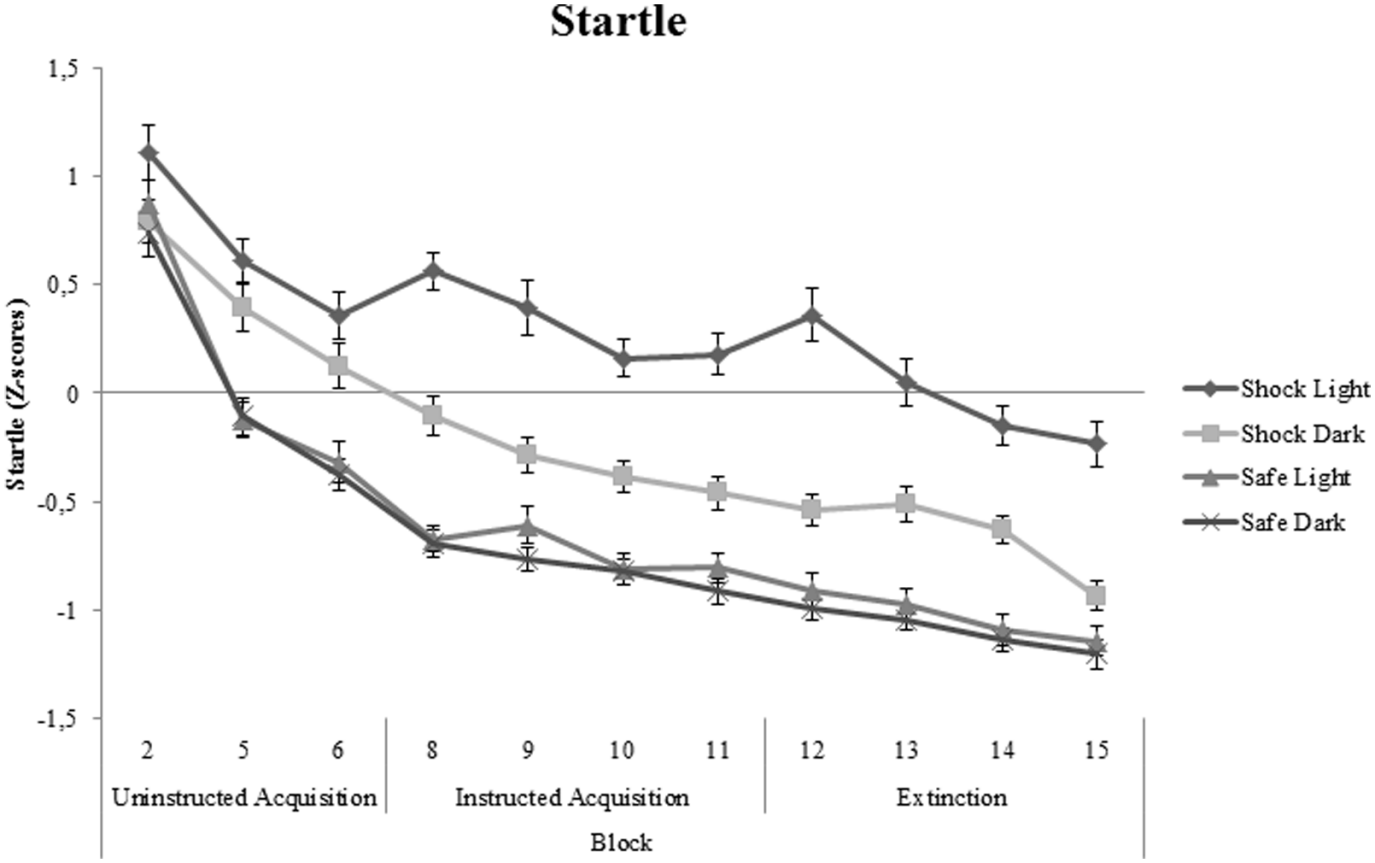
**Startle data (*Z*-scores) across all participants shown separately for shock room light, shock room dark, safe room light, and safe room dark**. No significant differences between treated anxiety patients and control subjects were found. Error bars show ±1 SEM.

Significant cue-specific FPS (shock room light vs. shock room dark), context-specific FPS (shock room dark vs. safe room dark) and generalization FPS (shock room light vs. safe room light) were demonstrated across subjects during uninstructed acquisition, instructed acquisition and extinction, as shown in **Table 3**. These main effects of cue, context and generalization support the validity of our conditioning paradigm. Startle data across participants are shown in **Figure 3**.

**Table 3:** Main effects of cue, context and generalization: startle data and subjective fearfulness ratings.

		df	*F*	*p*	$\eta_{p}^{2}$	Observed power
**Fear potentiated startle**						
Uninstructed acquisition	Cue	1, 40	6.5	0.015	0.139	0.70
	Context	1, 41	18.6	0.001	0.31	0.99
	Generalization	1, 40	34.6	0.001	0.464	1.00
Instructed acquisition	Cue	1, 41	90.5	0.001	0.69	1.00
	Context	1, 41	79.2	0.001	0.66	1.00
	Generalization	1, 41	125.8	0.001	0.754	1.00
Extinction	Cue	1, 41	88.7	0.001	0.684	1.00
	Context	1, 41	48.1	0.001	0.540	1.00
	Generalization	1, 41	123.9	0.001	0.751	1.00
**Subjective fearfulness**						
Uninstructed acquisition	Cue	1, 41	5.0	0.030	0.11	0.59
	Context	1, 41	35.4	0.001	0.46	1.00
	Generalization	1, 41	43.3	0.001	0.51	1.00
Instructed acquisition	Cue	1, 41	31.2	0.001	0.43	1.00
	Context	1, 41	25.2	0.001	0.38	1.00
	Generalization	1, 41	61.3	0.001	0.60	1.00
Extinction	Cue	1, 41	19.6	0.001	0.32	0.99
	Context	1, 41	23.2	0.001	0.36	1.00
	Generalization	1, 41	52.4	0.001	0.56	1.00

#### Startle Amplitudes During Acquisition

There were no significant differences between treated anxiety patients and control subjects in cue-specific FPS during both the uninstructed, *F*(1,33) = 0.1, *p* = 0.923, $\eta_{p}^{2}=0.01$, and instructed acquisition phase, *F*(1,34) = 0.1, *p* = 0.944, $\eta_{p}^{2}=0.01$. Context-specific FPS was neither significantly different in treated anxiety patients compared to control subjects during uninstructed, *F*(1,34) = 0.1, *p* = 0.863, $\eta_{p}^{2}=0.01$, and instructed acquisition of fear, *F*(1,34) = 2.4, *p* = 0.132, $\eta_{p}^{2}=0.07$. In addition, there were no significant patient-control differences in generalization FPS during the uninstructed, *F*(1,33) = 0.8, *p* = 0.383, $\eta_{p}^{2}=0.02$, and instructed acquisition phase, *F*(1,34) = 0.1, *p* = 0.739, $\eta_{p}^{2}=0.01$. These non-significant differences in fear acquisition between treated anxiety patients and control subjects imply that it is not likely that potential group differences in fear extinction are confounded by pre-existing group differences.

Results from the main analysis demonstrated a significant interaction between covariate BAI, generalization FPS (shock room light vs. safe room light), and block during instructed acquisition, *F*(2.9,98.8) = 4.0, *p* = 0.010, $\eta_{p}^{2}=0.11$. Follow up analyses demonstrated a significant increase of generalization FPS between early instructed and late instructed acquisition in subjects with low anxiety symptoms at time of participation, *F*(1,18) = 5.1, *p* = 0.036, $\eta_{p}^{2}=0.22$. This effect was marginally significant in subject with high anxiety symptoms, *F*(1,16) = 3.9, *p* = 0.064, $\eta_{p}^{2}=0.20$. There was no significant difference between subjects with high vs. low current anxiety symptoms on generalization FPS during early (*F* = 0.330, *p* = 0.569) or late instructed acquisition (*F* = 0.278, *p* = 0.601). See also Supplementary Figure S1 for the startle data, shown separately for subjects with high and low anxiety symptoms.

#### Startle Amplitudes During Extinction

From late acquisition to late extinction, no significant differences were found between treated anxiety patients and control subjects in cue-specific FPS, *F*(1,34) = 0.3, *p* = 0.606, $\eta_{p}^{2}=0.01$, context-specific FPS, *F*(1,34) = 0.3, *p* = 0.575, $\eta_{p}^{2}=0.01$, and generalization FPS, *F*(1,34) = 0.1, *p* = 0.766, $\eta_{p}^{2}=0.01$. However, covariate BAI did significantly interact with cue-specific FPS, context-specific FPS and generalization FPS. These interaction effects, as well as subsequent follow up analyses, are described below per contrast (see also Supplementary Figure S1).

##### Cue-specific FPS

**FIGURE 4:**
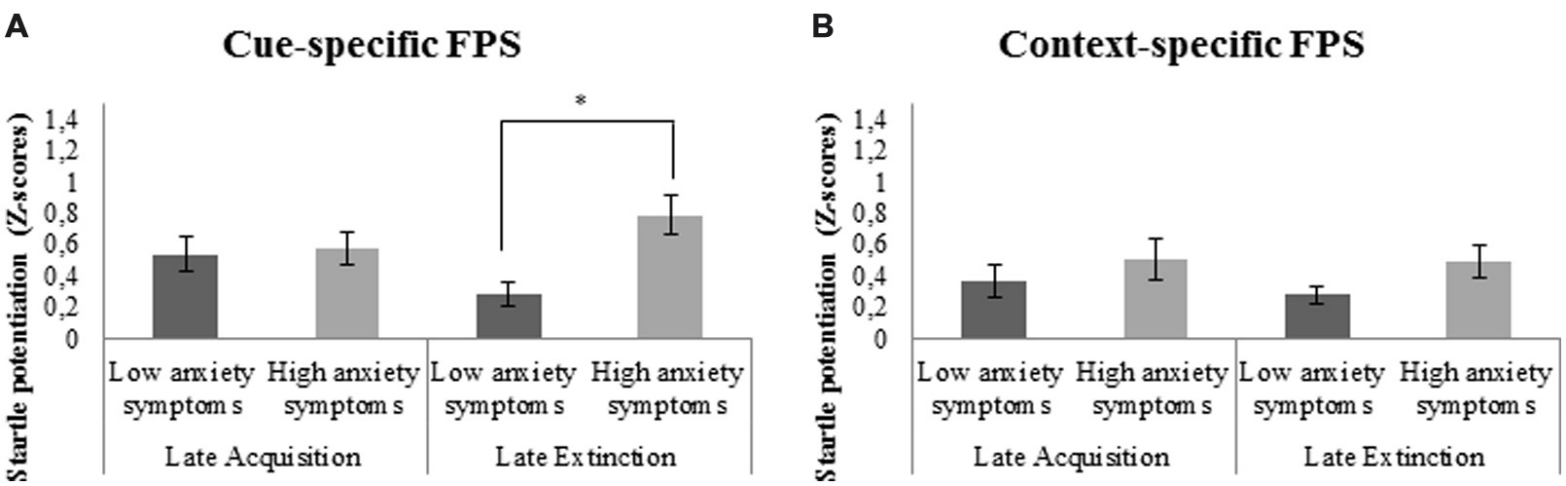
**(A)** Significant stronger cue-specific fear-potentiated startle (FPS; shock room light vs. shock room dark) during late extinction in subjects with high as compared to low anxiety symptoms. **(B)** No significant differences in context-specific FPS (shock room dark vs. safe room dark) between subjects reporting high vs. low anxiety symptoms over the past week. Error bars show ±1 SEM. ^∗^*p* < 0.05.

There was a significant 3-way interaction between cue-specific FPS, block, and covariate BAI. Follow-up analyses with BAI as between-subjects factor demonstrated significantly higher cue-specific FPS in subjects with high compared to low anxiety symptoms during late extinction, *F*(1,35) = 13.0, *p* = 0.001, $\eta_{p}^{2}=0.27$; **Figure 4A**. There was no significant reduction of cue-specific FPS throughout extinction (from late acquisition to late extinction) in subjects reporting low and high anxiety symptoms over the past week, *F*(1,18) = 2.7, *p* = 0.116, $\eta_{p}^{2}=0.13$ and *F*(1,16) = 4.0, *p* = 0.06, $\eta_{p}^{2}=0.20$, respectively.

##### Context-specific FPS

Main analyses demonstrated a significant interaction between context-specific FPS and covariate BAI. However, no significant differences in context-specific FPS were found during late extinction between those subjects reporting high vs. low anxiety symptoms, *F*(1,35) = 2.3, *p* = 0.137, $\eta_{p}^{2}=0.06$ (**Figure 4B**). There was also no significant decrease of context-specific FPS throughout extinction in subjects with low, *F*(1,18) = 1.42, *p* = 0.249, $\eta_{p}^{2}=0.07$, or high anxiety symptoms, *F*(1,16) = 0.04, *p* = 0.842, $\eta_{p}^{2}<0.01$.

##### Generalization FPS

**FIGURE 5:**
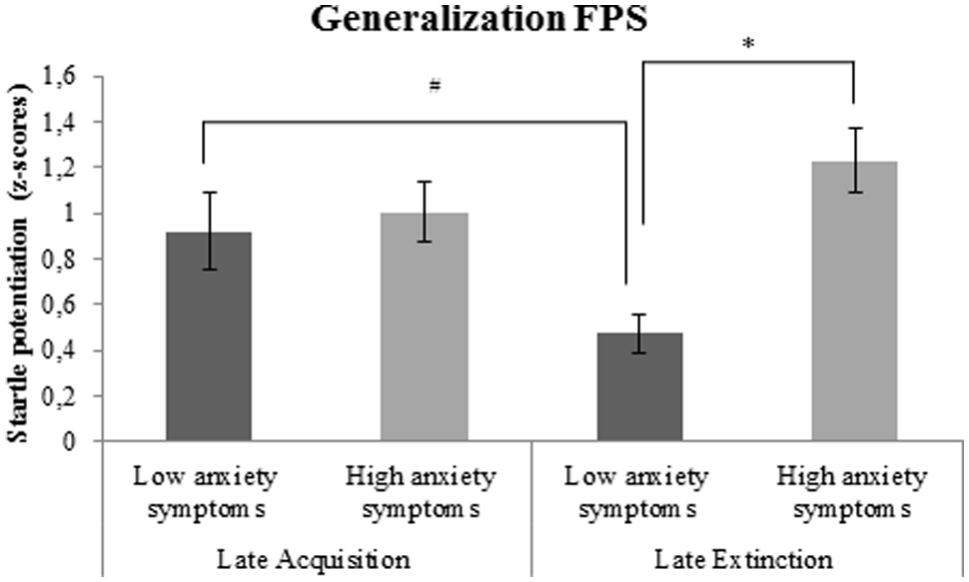
**Generalization FPS (shock room light vs. safe room light) in subjects with high vs. low anxiety symptoms**. Error bars show ±1 SEM. ^∗^*p* < 0.05; #*p* = 0.051.

There was a significant interaction between generalization FPS and covariate BAI. Follow-up analyses, with BAI included as between-subjects factor, demonstrated significantly higher generalization FPS during late extinction in subjects with high compared to low anxiety symptoms at the time of participation, *F*(1,35) = 23.3, *p* < 0.001, $\eta_{p}^{2}=0.40$; **Figure 5**. Furthermore, there was a marginally significant reduction of generalization FPS from late acquisition to late extinction in subjects with low anxiety symptoms, *F*(1,18) = 4.4, *p* = 0.051, $\eta_{p}^{2}=0.20$, but not in subjects with high anxiety symptoms, *F*(1,16) = 1.7, *p* = 0.210, $\eta_{p}^{2}=0.10$.

### Subjective Fearfulness Ratings

Significant main effects of cue (shock room light vs. shock room dark), context (shock room dark vs. safe room dark) and generalization (shock room light vs. safe room light) were demonstrated during uninstructed acquisition, instructed acquisition and extinction across subjects, as shown in **Table 3**.

**FIGURE 6:**
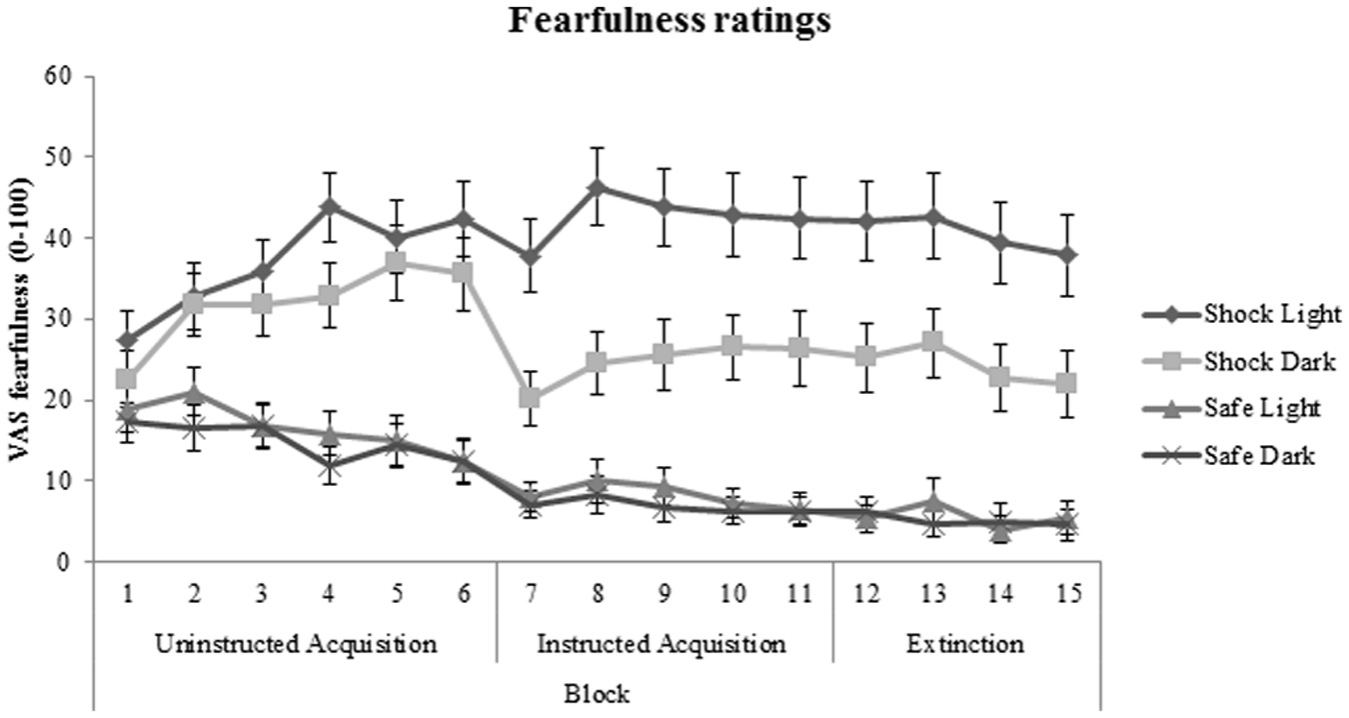
**Fear acquisition and extinction of subjective fearfulness shown across all participants and displayed separately for shock room light, shock room dark, safe room light, and safe room dark**. No significant differences in subjective fearfulness were found between treated anxiety patients and control subjects. Error bars show ±1 SEM.

No significant patient-control differences were found in cue-specific, context-specific, or generalization contrasts of VAS fearfulness ratings during acquisition and extinction phases (all *p* values > 0.50). In addition, medication use (between-subjects factor) and BAI (covariate) did not significantly interact with cue-specific FPS/context-specific FPS or generalization FPS. Subjective fearfulness ratings across all participants are shown in **Figure 6**.

## Discussion

The current study aimed to explore differences in fear extinction between treated anxiety disordered patients and healthy control subjects. Specifically, the current explorative study aimed to gain more insight in whether impaired fear extinction is present in patients who have been treated for an anxiety disorder in the past.

Results demonstrated a significantly stronger baseline startle reflex in treated anxiety patients as compared to control subjects during the habituation phase, which is in line with earlier findings in patients with panic disorder (Ludewig et al., 2005). Based on previously demonstrated patient-control differences in the extinction of fear (Duits et al., 2015) and based on the relative stability of extinction parameters (Fredrikson et al., 1993; Hettema et al., 2003; Zeidan et al., 2012; Torrents-Rodas et al., 2014), we hypothesized that patients who have been treated for an anxiety disorder in the past would display stronger fear responses to threat cue and context, and stronger fear generalization during extinction as compared to healthy controls. The present (startle and subjective fearfulness) data do not support this hypothesis, as no differences in the rate of fear extinction and the expression of fear during extinction were found between treated anxiety patients and control subjects.

However, FPS results demonstrated an association between high anxiety symptoms over the past week (as measured using the BAI) and impaired fear extinction across subjects. More specifically, during late extinction, subjects with high anxiety symptoms displayed stronger (cue-specific and generalization) FPS as compared to those with low anxiety symptoms. This finding reflects that during extinction, in which the cue does not predict the occurrence of danger anymore, high levels of anxiety symptoms are associated with stronger fear responses to the threat cue. These results suggest that fear extinction is predominantly associated with anxiety symptoms directly preceding or at the time of testing, and, in line with a dimensional approach (RDoC initiative; Insel et al., 2010), is not restricted to previous or current anxiety disorder diagnosis. Furthermore, the finding that BAI, but not a diagnosis of an anxiety disorder in the past, is significantly associated with fear extinction suggests that even though fear extinction is relatively stable over time and partly determined by genetics (Fredrikson et al., 1993; Hettema et al., 2003; Zeidan et al., 2012; Torrents-Rodas et al., 2014), the circuitry underlying fear extinction is dynamic. Meta-analyses have shown significant differences between individuals with a current anxiety diagnosis and control subjects (Lissek et al., 2005; Duits et al., 2015), but the current findings may reflect plasticity due to new learning experiences, especially in the context of an exposure therapy. Due to the similarity of fear extinction to processes putatively underlying exposure therapy (Massad and Hulsey, 2006; Hofmann, 2008; Myers and Davis, 2008), fear extinction performance might be improved by exposure therapy, the treatment of choice in anxiety disorders. Neuroimaging studies provide evidence for stronger activation of the ventromedial prefrontal cortex, an area important in extinction, after just one session of exposure therapy (Schienle et al., 2007). Results from exploratory analyses in the current study demonstrated no significant difference in fear conditioning between treatment responders and non-responders. However, the time between treatment and participation in the conditioning paradigm had been variable, and sample sizes were small. Furthermore, data from the current study did not allow the examination of changes in fear extinction before and after treatment, as no pre-treatment measurement of fear extinction was included. Therefore, further conclusions must await a prospective study that includes pretreatment fear extinction measures to determine whether fear extinction is improved by successful exposure therapy. Nevertheless, the present results allow the conclusion that the current anxiety status seems to have more impact on fear extinction mechanisms than a past anxiety diagnosis.

Subjective fearfulness ratings did not show a similar pattern, demonstrating no differences in fear extinction between subjects reporting high vs. low anxiety symptoms. A recent meta-analysis found no difference in sensitivity of physiological vs. subjective outcome measures to fear conditioning and extinction abnormalities in anxiety patients vs. controls (Duits et al., 2015). Yet, in this study the FPS outcome parameter was sensitive to individual differences in current anxiety symptoms, while subjective ratings were not. This is in line with numerous studies in which pharmacological manipulations or genetic analyses demonstrate effects on physiological parameters in absence of effects on subjective reports (Kindt et al., 2009; Soeter and Kindt, 2011; Heitland et al., 2012, 2013; Klumpers et al., 2012; Baas and Heitland, 2014). This may reflect a closer relationship between physiological parameters (relative to subjective reports) and the neurobiological circuitry that is affected, while subjective reports are more susceptible to cognitive evaluation and experimenter demand (Boddez et al., 2013).

Caution is required when interpreting the current findings, as several limitations should be taken into account. First, the current study did not control for potential differences in fear extinction between anxiety patients and controls prior to exposure therapy, as differences were only investigated after patients had received treatment. We therefore recommend to include both pre- and post-treatment measurements of extinction in future studies. Second, replication of the current study is recommended in a larger sample, thereby allowing to examine differences across diagnostic groups (patients with panic disorder vs. social anxiety). So far, our meta-analysis (Duits et al., 2015) and recent findings from our group (Duits et al., in preparation) found no support for significant differences in fear conditioning across diagnostic groups. Third, the conditioning procedure would benefit from the inclusion of at least one additional CS in both contexts that is never paired with the US, to measure generalization effects and to further specify the effects of cue conditioning. Fourth, we did not formally test hearing acuity and drug use in participants.

## Conclusion

No significant differences in fear extinction were found between treated anxiety patients and healthy controls, but stronger generalization and cue-specific fear potentiation were demonstrated during late extinction in subjects with high compared to low anxiety symptoms over the past week. Together, these results may suggest that current anxiety symptoms rather than past anxiety disorder diagnosis may play an impairing role in fear extinction processes. This should be taken into account in future fear conditioning paradigms. The anxiety symptoms as assessed with the BAI may represent a dimensional variable that relates closely to fear extinction and that cuts across different diagnostic categories within the anxiety disorder spectrum. In addition, future prospective longitudinal studies are needed to shed light on the course of individual differences in fear extinction and to further investigate whether persistence of impaired fear extinction is a risk factor that mediates future relapse in anxiety disordered patients after treatment.

## Author Contributions

JB and IH contributed to the study design. Testing and data collection was performed by PD. Data-analysis was performed by PD, under the supervision of JB, DC, and IH; PD drafted the paper, and DC. JB and IH provided critical revision. All authors approved the final version of the paper for submission.

## Conflict of Interest Statement

The authors declare that the research was conducted in the absence of any commercial or financial relationships that could be construed as a potential conflict of interest.
