# Supplementary material for: High Current Anxiety Symptoms, But Not a Past Anxiety Disorder Diagnosis, are Associated with Impaired Fear Extinction
Source: Front Psychol. 2016 Feb 26;7:252. doi: 10.3389/fpsyg.2016.00252 (PMC4767935; doi:10.3389/fpsyg.2016.00252)
Supplement: Supplementary file 1 [file Data_Sheet_1.DOCX]

***Supplementary Material***

**High current anxiety symptoms, but not a past anxiety disorder diagnosis, are associated with impaired fear extinction.**

**Puck Duits^1,2*^, Danielle C. Cath^1,2^, Ivo Heitland^3,4^ and Johanna M.P. Baas^3,4^**

*** Correspondence:** Puck Duits: p.duits@uu.nl

**Supplementary table 1.** Psychiatric comorbidity in the patient group at pre-treatment

| Psychiatric Comorbidity | Patient group (N = 26) | |
| --- | --- | --- |
|  | N |  |
| Past major depressive disorder | 9 |  |
| Current dysthymic disorder | 8 |  |
| Recurrent major depressive disorder | 5 |  |
| Specific phobia | 5 |  |
| Generalized anxiety disorder | 2 |  |
| Hypochondriasis | 2 |  |
| Panic disorder with agoraphobia | 2 |  |
| Current major depressive disorder  Posttraumatic stress disorder | 2  2 |  |
| Obsessive compulsive disorder | 1 |  |

Shock expectancy ratings

Shock expectancy ratings were obtained using forced choice questions that were simultaneously presented with two screenshots of different conditions. Three distinct contrasts were used, each made up of two conditions: shock context light versus shock context dark, shock context dark versus safe context dark, safe context light versus safe context dark. All contrasts were presented three times after every block. Within the shock expectancy ratings, participants had to indicate which of the two presented screenshots represented the highest risk of receiving a shock. Participants could either choose one of the conditions, or indicate that the risk was equal in both conditions. Next, participants were classified as cue aware versus unaware, and context safety aware versus unaware based on the expectancy ratings during the uninstructed acquisition phase. A participant was classified as cue aware in case a correct answer was given in at least 50 % of cases for the contrast shock room light versus shock room dark. The classification of context contingency awareness was based on the contrast of shock room dark versus safe room dark. As in a previous study, acquisition of context conditioning could be deduced in two ways, depending on cue awareness (Baas, 2013). First, context conditioning could be deduced when subjects indicated the risk to be highest in the shock room in the dark in at least 50 % of the presentations of this contrast during the uninstructed acquisition phase. However, after acquisition of the cue contingency, a subset of participants indicated correctly that the risk of receiving a shock was equal in both the shock room dark and the safe room dark. After all, in absence of cue awareness, the shock context was the only predictive variable for onset of the shock, but may no longer be seen as a predictor after learning the cue contingency. To be classified as context safety aware, subjects must have satisfied the criterion of cue awareness. In addition they must have given a minimum of three out of four correct answers (chance of shock is equal in the shock and safe contexts in the dark) during the last two blocks of the uninstructed acquisition phase. According to these criteria of cue and context safety (un)awareness, participants were assigned to one out of four groups: 1) cue and context safety aware 2) cue but not context safety aware 3) cue unaware 4) cue and context unaware. As in previous studies, participants were excluded from further analyses when they were classified as cue and context unaware (Baas, 2013), as the absence of any learning about contingencies, even the easy to learn context contingency, questions whether participants have understood the task and/or the subjective assessment scales.

Results shock expectancy ratings

At the end of the uninstructed acquisition phase, 57 % of all participants (N = 26) failed to satisfy the criteria for cue contingency, and were labeled as ‘cue unaware’ (supplementary table 2). The failure of learning the cue-contingency in a substantial number of participants may have derived from the use of relatively low reinforcement rates and changing between two different partial reinforcement schedules between blocks (37.5 % versus 75 %). Criteria for cue contingency were met by 37 % of all participants (N = 17). However, the majority of these cue aware participants (N = 14) failed to identify safety of the context and were therefore categorized as ‘cue but not context safety aware’. The remaining three cue aware participants met the criteria for being ‘cue and context safety aware’. Lastly, three healthy control subjects failed to demonstrate both cue and context awareness and were excluded from the analyses. Exclusion of these subjects did not significantly alter the results of the current study. The final sample then comprised 26 treated patients and 17 healthy control subjects. Chi-square tests indicated no differences in awareness between the patient and control group.

**Supplementary table 2.** Classification of cue and context safety awareness in the patient group and control group

|  | Patient group | | Control group | | Whole sample | |
| --- | --- | --- | --- | --- | --- | --- |
|  | N | % | N | % | N | % |
| Cue unaware | 17 | 65.4 | 9 | 45 | 26 | 56.5 |
| Cue but not context safety aware | 8 | 30.8 | 6 | 30 | 14 | 30.5 |
| Cue and context safety aware | 1 | 3.8 | 2 | 10 | 3 | 6.5 |
| Cue unaware and context unaware | 0 | 0 | 3 | 15 | 3 | 6.5 |

**Supplementary table 3.** Classification of clinically relevant anxiety symptoms (based on cutoff scores from the body sensations questionnaire and social phobia and anxiety inventory) in treated patients and controls with low versus high current anxiety symptoms.

|  | Patient group | | Control group | |
| --- | --- | --- | --- | --- |
|  | Low BAI | High BAI | Low BAI | High BAI |
| Below cut-off | 6 | 4 | 10 | 3 |
| Above cut-off | 4 | 11 | - | - |

**
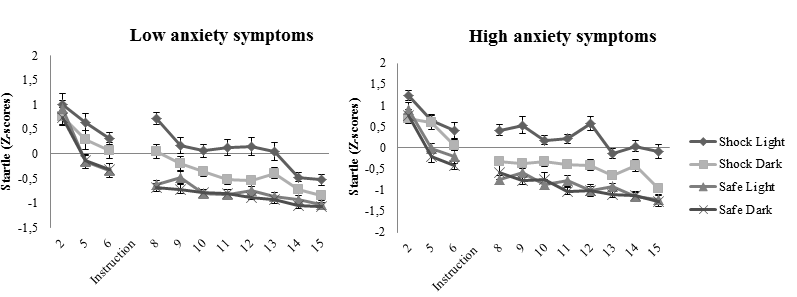
**

**Supplementary figure 1.** Startle data are shown across blocks and displayed separately for subjects with low anxiety symptoms (left graph) and subjects with high anxiety symptoms (right graph). Error bars show ± 1 standard error of the mean.

References

Baas, J. M. P. (2013). Individual differences in predicting aversive events and modulating contextual anxiety in a context and cue conditioning paradigm. *Biological Psychology, 92*(1), 17-25.
